# Fertility-Sparing Approach in Patients with Endometrioid Endometrial Cancer Grade 2 Stage IA (FIGO): A Qualitative Systematic Review

**DOI:** 10.1155/2022/4070368

**Published:** 2022-09-27

**Authors:** Pierluigi Giampaolino, Valeria Cafasso, Dominga Boccia, Mario Ascione, Antonio Mercorio, Francesco Viciglione, Mario Palumbo, Paolo Serafino, Cira Buonfantino, Maria Chiara De Angelis, Paolo Verrazzo, Giovanna Grasso, Giuseppe Gullo, Giuseppe Bifulco, Luigi Della Corte

**Affiliations:** ^1^Department of Public Health, University of Naples Federico II, 80131 Naples, Italy; ^2^Department of Neuroscience, Reproductive Sciences and Dentistry, School of Medicine, University of Naples Federico II, 80131 Naples, Italy; ^3^PO San Giuseppe Moscati of Aversa-ASL Caserta, Italy; ^4^Department of Obstetrics and Gynecology, AOOR Villa Sofia Cervello, IVF Public Center, Palermo, Italy

## Abstract

**Background:**

Endometrial cancer (EC) is one of the most common gynecologic malignancy, mostly in postmenopausal women. The gold standard treatment for EC is surgery, but in the early stages, it is possible to opt for conservative treatment. In the last decade, different clinical and pathological markers have been studied to identify women who respond to conservative treatment. A lot of immunohistochemical markers have been evaluated to predict response to progestin treatment, even if their usefulness is still unclear; the prognosis of this neoplasm depends on tumor stage, and a specific therapeutic protocol is set according to the stage of the disease.

**Objective:**

(1) To provide an overview of the conservative management of Stage 1A Grade (G) 2 endometrioid EC (FIGO) and the oncological and reproductive outcomes related; (2) to describe the molecular alterations before and after progestin therapy in patients undergoing conservative treatment.

**Materials and Methods:**

A systematic computerized search of the literature was performed in the main electronic databases (MEDLINE, Embase, Web of Science, PubMed, and Cochrane Library), from 2010 to September 2021, in order to evaluate the oncological and reproductive outcomes in patients with G2 stage IA EC who ask for fertility-sparing treatment. The expression of several immunohistochemical markers was evaluated in pretreatment phase and during the follow-up in relation to response to hormonal therapy. Only scientific publications in English were included. The risk of bias assessment was performed. Review authors' judgments were categorized as “low risk,” “high risk,” or “unclear risk” of bias.

**Results:**

Twelve articles were included in the study: 7 observational studies and 5 case series/reports. Eighty-four patients who took progestins (megestrol acetate, medroxyprogesterone acetate, and/or levonorgestrel-releasing intrauterine devices) were analyzed. The publication bias analysis turned out to be “low.” 54/84 patients had a complete response, 23/84 patients underwent radical surgery, and 20/84 had a relapse after conservative treatment. Twenty-two patients had a pregnancy. The length of follow-up was variable, from 6 to 142 months according to the different studies analyzed. Several clinical and pathological markers have been studied to identify women who do not respond to conservative treatment: PR and ER were the most studied predictive markers, in particular PR appeared as the most promising; MMR, SPAG9, Ki67, and Nrf2-survivin pathway provided good results with a significant association with a good response to progestin therapy. However, no reliable predictive markers are currently available to be used in clinical practice.

**Conclusions:**

The conservative treatment may be an option for patients with stage IA G2 EEC who desire to preserve their fertility. The immunohistochemical markers evaluation looks promising in predicting response to conservative treatment. Further large series and randomized clinical trials are needed to confirm these results.

## 1. Introduction

Endometrial carcinoma (EC) is the most common gynecologic malignancy in developed countries, with an estimated worldwide incidence of 382,069 new cases per year [1, 2]. Furthermore, global estimates show rising incidence rates both in developed and developing countries, especially for an increased prevalence of risk factors such as obesity [3–5]. Patient age affects both EC incidence and mortality [6–8]; more than 90% of cases of EC occur in perimenopause women, and 14-25% are in premenopausal [9, 10] with the median age at diagnosis of 61 years [11], and the survival rate declines with age [12]. Historically, EC has been classified into two main clinic-pathological and molecular types: (1) Type I, the estrogen-dependent endometrioid-type endometrial cancer (EEC), is the most common histotype of EC [13]; (2) type II, the nonendometrioid subtypes that include serous, clear-cell, undifferentiated carcinomas, and malignant mixed Mullerian tumors, is typically associated with old age, high stage, and advanced grade and poor prognosis [14, 15]. Tumor grade and myometrial invasion increase with age, accounting in part for the considerably worse prognosis of older patients [16, 17]. In the last years, it has been demonstrated that specific molecular features have a major prognostic value in endometrial carcinoma. In 2013, another classification has been drawn up by the Cancer Genome Atlas (TCGA) that stratified endometrial carcinoma into four prognostic molecular subgroups: POLE/ultramutated (POLE); microsatellite-instable/hypermutated (MSI); copy-number-low/TP53-wild-type (CNL), conceptually similar to “type I” endometrial carcinoma; and copy-number-high/TP53-mutant (CNH), which is similar to “type II” endometrial carcinoma, particularly to serous subtypes [18]. These recent advances have expanded our understanding of the genomic features of ECC, leading to the identification of molecular signatures predictive of individual tumor behavior. In detail, the *POLE ultramutated* tumors show the most favorable prognosis as well as it is associated with longer progression-free survival. Usually, this group is linked to endometrioid histotype of EC and related to the following mutated genes: POLE, PTEN, PIK3R1, PIK3CA, FBXW7, KRAS, and TP53 [19]. The *hypermutated type* with microsatellite instability (MSI)/mismatch repair deficient (MMRd) group is characterized by an inactivation due to mutation accumulations called MSI through several mechanisms: insertions, deletions, point mutations, loss of heterozygosity, copy number changes, structural rearrangements, and methylation of a promoter gene. This group is characterized by PTEN, KRAS, and ARID1A mutations, linked to intermediate prognosis, and also found in the endometrioid EC [19, 20]. The third group is the *low copy number* characterized by an intermediate prognosis and also associated with endometrioid EC: in this group CTNNB1 and PTEN are mutated, and the genome mutations are due to duplication or deletion that alters the number of DNA base pairs. Finally, the *high copy number* is characterized by an unfavorable prognosis and linked to serous histotype of EC. In this case, genomic instability of the tumor and fast growth progression and invasion are typical. The mutated genes are TP53, FBXW7, and PPP2R1A.

Following the characterization of the molecular EC classification, there is a growing interest towards the identification of risk factors and stratification of women based on the molecular biology of the disease.

Established risk factors include age (>55 years) and hyperestrogenic status associated with nulliparity, early menarche, late age at menopause, ovarian disease, therapy with tamoxifen, chronic liver disease, obesity, and metabolic syndrome. Obesity is associated with peripheral estrogen conversion via aromatization in adipose tissue [21, 22]. Although immunohistochemical and molecular examinations can be performed on endometrial biopsy, due to identifying preventively the responders to the conservative treatment, avoiding the risk of disease progression resulting from ineffective therapy, nowadays no reliable predictive markers are currently available to be used in the clinical practice. In women candidates for conservative treatment, operative hysteroscopy could be advantageous to provide samples allowing complete assessment.

Currently the gold standard treatment of EC is surgery: total hysterectomy (TH) with bilateral salpingo-oophorectomy (BSO), peritoneal cytology, and lymph node dissection have excellent survival outcomes, particularly for low-grade endometrioid tumors, but are not ideal for women interested in future fertility [23]. Although aggressive interventions should be considered to treat high-grade EC, a conservative approach should be taken into consideration for women wishing to become pregnant. Nowadays, guidelines for the conservative management of endometrial cancer focus primarily on grade (G)1 stage IA, while G2 is not yet included. However, relatively few fertility-saving studies cover G2. Although the degree varies, the conservative treatments are similar. In particular, fertility-sparing treatment approaches for patients who wish to preserve childbearing involve orally administrated progestin together with hysteroscopic ablation of lesions [24–26]. The most used and effective endocrine treatments with oral progestins include medroxyprogesterone acetate (MPA, 400–600 mg/day) or megestrol acetate (MA, 160–480 mg/day). More recently, levonorgestrel-releasing intrauterine devices (LNG-IUDs) have been used. These devices have been proved to be the most effective among the available progestins [27, 28]. There are several eligibility criteria to use this treatment in EC: endometrioid-type, low-grade, no myometrial invasion (stage IA), no lymphovascular space invasion, and no cervical or adnexal involvement [29, 30].

Various clinical and pathological markers have been studied to identify women who will not respond to hormonal treatment, but their usefulness is still unclear: PR and ER were the most studied markers because PR is involved in the pathogenesis of EH, just like Nrf2 and AKR1C1 who supported the resistance to progestogens. Ki67 antigen is a nuclear protein that represents a useful marker of the cell population growth. SPAG9 (sperm associated antigen 9) is a protein that promotes the switching of protein kinases and their transcription factor targets for the activation of specific signaling pathways. Mismatch repair (MMR) proteins, MLH-1, MSH-2, MSH-6, and PMS-2, are a system to recognize and repair erroneous insertion, deletion, and misincorporation of bases that can arise during DNA replication and recombination, as well as repair some forms of DNA damage. Consequently, its alteration is associated with tumor transformation. PTEN and DUSP6 are enzymes of the phosphatase class that participate in a transduction pathway of the intracellular signals, which regulate the cell cycle, limiting cell division and initiating cells towards apoptosis. In this way, these signals prevent uncontrolled cell growth that can lead to the onset of tumors.

However, no reliable predictive markers of progestogens resistance are currently available to be used in clinical practice, so it will be necessary to study them further [31, 32].

The studies in literature focus on atypical endometrial hyperplasia (AEH) and EEC, and there are little data about Grade G2 EEC. The aim of this systematic review is to provide an overview of conservative management of EEC G2 Stage 1A and to analyze the oncological and reproductive outcomes related, reporting the molecular alterations before and after conservative treatment in patients treated with hormonal therapy.

## 2. Materials and Methods

### 2.1. Data Sources

The research protocol was designed a priori, defining methods for searching the literature. The research was conducted using the following electronic databases, MEDLINE, Embase, Web of Science, Pub Med, and Cochrane Library. The studies were identified with the use of a mesh combination of the following keywords: “fertility sparing treatment,” “endometrial cancer,” “stage IA,” “grade 2,” “conservative treatment,” “endometrioid type,” “low grade,” “progestin,” “molecular markers,” and “immunohistochemistry” from 2010 to September 2021. Two authors (LDC and VC) independently screened titles and abstracts of studies obtained in the search. All types of studies were selected, and each potentially relevant study was obtained in full text and assessed for inclusion independently by the authors. Disagreements were resolved by consensus with a third reviewer (DB). All references of the retrieved studies were also reviewed to avoid missing relevant publications. Only scientific publications in English were included. All reports related to experimental studies conducted on in vitro or animal models were excluded from the analysis. Proceedings of scientific meetings and abstracts were not considered.

### 2.2. Study Selection and Risk of Bias

All articles describing fertility-sparing approach applied to stage IA G2 EEC patients were considered for review. Only original papers that reported specific experience data on the topic were included. Relevant aspects of every article were recorded and commented, with particular attention to the modality of treatment applied and described outcomes. Fertility-sparing therapy include well-established algorithm such as continuous progestin-based therapy: MA, MPA, or LNG-IUD with endometrial sampling every 3–6 months (either D&C or endometrial biopsy). To describe the strength and the level of evidence of the results, we applied the recent levels of evidence published by the NCCN Guidelines Version 1.2018 Uterine Neoplasms. All randomized and nonrandomized studies were included in our systematic review to analyze the expression of several immunohistochemical markers on endometrial biopsies in pretreatment and during the follow-up, and the association between this expression and the outcome of hormonal therapy. The risk of bias assessment was performed with Joanna Brigg's Critical Appraisal Tool for Case-Series. Review authors' judgments were categorized as “low risk,” “high risk,” or “unclear risk” of bias [33].

## 3. Results

**Figure 1:**
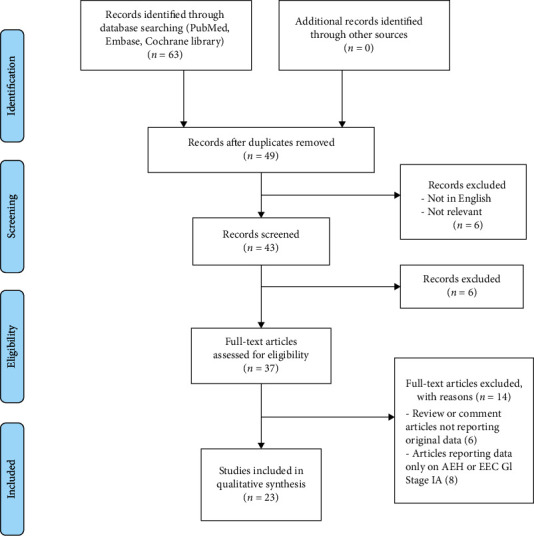
Flow diagram of systematic review search.

Figure 1 illustrates the selection of studies for inclusion in the systematic review. From the bibliographic search, a total of 63 articles were retrieved. Forty-three articles remained after title screening. Thirty-seven articles were evaluated for eligibility after abstract screening. Finally, 23 studies were included in the systematic review [34–45].

Of the 23 articles included in this review, 12 were used to provide an overview of the conservative management of Stage 1A Grade (G) 2 EEC (FIGO) and the oncological and reproductive outcomes related; of these, 5 are retrospective observational studies [34–38], 4 case reports (one paper reporting 3 cases and another one reporting 2) [39–42], 1 a retrospective case series [43], 1 a prospective study [44], and 1 a multicenter retrospective cohort study [45]. The remaining analysis focused on immunohistochemical markers, although the data extrapolation was not easy due to the considerable heterogeneity of the studies published up to now. According to current published English papers, 84 patients with G2 stage IA took part in fertility-sparing treatment. The youngest patient was 13 years old as reported by Kim et al. [42], while the oldest was 85.2 years old by Pal et al. [43]. Pal et al. considered a wide age range, 18.5-85.2 years old, because they also included patients affected by obesity or with an important anesthetic risk that would have contraindicated surgery. The characteristics of the included patients are summarized in Table 1.

Regarding treatments, 16/84 patients analyzed were treated with MPA and LNG-IUDs [34, 35], 29 patients only with LNG-IUD (but 12 cases analyzed by Falcone et al. added the hysteroscopic resection to the LNG-IUDs) [36, 40, 43, 44], 14 patients only with MA (but in 2 cases analyzed by Shan, metformin was added to MA, and 4 cases analyzed by Falcone et al. saw the addition of the hysteroscopic resection to MA) [37–39, 41, 43], and 16 patients with MA and MPA [42, 45]. For more details about treatments, see Table 1.

The other 11 articles included in this review were used to describe the molecular alteration before and after conservative treatment in patients treated with hormonal therapy; of these, 10 are retrospective reviews [46–55], and 1 study is a prospective phase II trial [56].

For more details about immunohistochemical markers and their significant association, see Table 2.

To simplify the presentation, the results are divided into three sections based on oncological and reproductive outcomes, as well as follow-up (Table 3).

### 3.1. Quality Characteristics

Publication bias was considered to be “low” since only 4 studies out of 12 included case series or case reports. The inclusion of one prospective cohort study and the remaining retrospective studies decreased the paper's bias. A detailed quality assessment of the included studies is reported in Table 4.

#### 3.1.1. Oncological Outcomes

Out of a total of 84 patients (age range 13-85 years old), 54 had a complete response to hormonal therapy, 5 had a partial response, 6 had a stable disease, 7 had a disease progression, while the remaining ones were not reported by the authors. However, 23/84 patients underwent surgery: in particular, two had total hysterectomy with bilateral salpingo-oophorectomy, and the remaining had an unspecified hysterectomy with no mention of lymphadenectomy, while 20/84 had a relapse after the hormonal treatment in different periods from 6 to 142 months, according to the different study analyzed.

Regarding to seven patients with disease progression, there was tumor transition to a lesion of higher grade or clinically progressive disease including myometrial invasion, extrauterine disease, or to lymph nodes. Five of them were analyzed by Falcone et al. [44]: in particular 2 patients underwent definitive surgery, and the final pathology showed a FIGO2009 stage IA (with myometrial invasion) G3 endometrioid EC and a stage IA G1 endometrioid EC, respectively; another patient was suspected of cervical involvement and treated by definitive surgery with a diagnosis of stage II G2 endometrioid EC. In the second in last, an ovarian mass was found and treated by definitive surgery, showing a stage IA (with myometrial invasion) G1 endometrioid EC with a synchronous stage IC2 G2 endometrioid ovarian cancer (OC). The fifth patient received a diagnosis of G3 histology and myometrial invasion at the 9-month follow-up and underwent definitive surgery (stage IIIC1 G3 endometrioid EC).

Further two studies reported disease progression in two patients and then undergone radical surgery, caused by histological upgrade diagnosed during follow-up period [36, 37].

A detailed report of oncological outcomes is shown in Table 3.

#### 3.1.2. Reproductive Outcomes

After the hormonal therapy, 22 patients had a pregnancy: the majority of patients had a spontaneous pregnancy, while in 5 cases it was necessary to resort to an assisted reproduction technology (ART). Further women analyzed by Shan et al. have been trying spontaneous pregnancy and undergoing in vitro fertilization. About the live birth rate, the normal full-term deliveries were eight, but there were also four spontaneous first trimester miscarriages and one abortion. Unfortunately, in the analyzed studies, data on the cause of miscarriages/abortion are not reported.

The reproductive outcomes are shown in Table 3.

#### 3.1.3. Follow Up

The 84 patients analyzed by the included studies were followed up for a variable number of months, and in 59 cases, there was no evidence of disease, while two patients are still alive with disease. The data of remaining 23 patients is not known. The period of follow up for each case are shown in Table 3.

### 3.2. Molecular Marker Analysis

Eleven studies [46–56] with a total of 29 immunohistochemical markers were included in this review.

Only one study considered EC G2 [53], while all the others regarded AEH and EEC G1 [46–52, 56] or did not report the grade [54, 55].

Different immunohistochemical markers were analyzed and, when possible, also their expression levels with the response to the conservative treatment.

The treatment included MAP in most cases, followed by LNG-IUS.

Hormonal receptors were investigated in 6 studies [46–48, 51–53, 56].

Chung et al. evaluated the prognostic significance of the Proactive Molecular Risk Classifier for Endometrial Cancer molecular subtypes (mismatch repair deficiency, DNA polymerase epsilon mutation, wild-type p53, and abnormal p53) because it could be used as a predictive biomarker for selecting patients who could benefit from hormone therapy [53], while Van Gent et al. studied the progesterone antitumor effect in EEC by interacting with the Wnt and/or PI3K/Akt pathways and explored whether common activating genetic alterations in Wnt and PI3K/Akt signaling correlated with nonresponsiveness to progesterone therapy for low-grade EEC, but they found that these alterations did not predict resistance to progesterone treatment [52]. The morphological changes during the early stage of treatment or indices of proliferation, apoptosis, or hormone receptors have been investigated as reliable predictors of the hormonal response to uterus-preserving high-dose progestin therapy and established that a higher epithelial cell size ratio after 4 weeks of treatment could be a potential predictor of hormonal response [51]. About risk factors, Yang et al. established that obesity seems to be the most important for relapse after conservative treatment [47]. Regarding the use of LNG-IUD therapy, Westin et al. studied its activity in complex atypical hyperplasia and EEC G1 with a modest proportion demonstrating upfront progesterone resistance, and Reyes et al. examined hormone receptor expression levels and downstream gene activation in pretreatment and posttreatment with IUD biopsies as a biomarker for response to therapy and an indicator of PR function [48].

About the evaluated six studies, in five of them [46–48, 51, 53], both progesterone receptor (PR) and estrogen receptor (ER) were analyzed [56]. As regard the pretreatment, the association between PR level expression and the outcome was possible only in a single study [53], and an increase of PR was reported as a good response to the treatment (*p* = .011). In the other cases, no significant associations with the outcome of the progestogen-based therapy were found. In the follow-up, high-level expressions of ER and PRB (progesterone receptor type B) were associated with a poor response to conservative treatment, while low levels of the same markers were associated with a statistically significant good response [48].

Ki67 was analyzed in 3 studies [47, 51, 56]: in the pretreatment phase, high-level expression was associated with a poor response to the conservative treatment (*p* = .023) [56], while, during the follow-up, there was a relapse after conservative treatment in case of high-level expression of this marker (*p* = .033) [47].

Nrf2 (Nuclear factor erythroid 2-related factor 2) is an immunohistochemical marker investigated in 2 studies [54, 55], and there are results only in the follow-up, while in pretreatment, there is no significant association with the outcome of the progestogen-based therapy. In particular in both studies, high level of expression of Nrf2 was associated with a poor response to conservative treatment, but it was combined with survivin and AKR1C1 in 2 studies, respectively [54, 55], and also a high level of both these markers were associated with a poor response; however, the survivin expression was statistically significantly lower compared to not responders to conservative treatment (0.52 ± 0.03 vs. 8.52 ± 1, 25, *p* < .001, respectively). Also Nrf2 expression was significantly different among responders and not responders (0 vs. 5.12 ± 0.48, *p* < .001, respectively). No statistically significant differences among AEH and EC cases were reported [55].

SPAG9 (sperm-associated antigen 9) was analyzed in a single study [49], and the low level was associated with a good response (*p* = .005).

MLH1, MSH2, MSH6, and PMS2, known as MMR proteins, were analyzed in 2 studies [50, 53] and DDK3 in a single study [56]. In contrast with the last cases, there were significant results only in pretreatment where their low expressions were associated with a poor response.

No association with the outcome of the progestogen-based therapy was found for the other analyzed markers in particular ssDNA, FOXO1, PTEN, beta catenin, p53, EIG121, IGF1/2, IGFBP1, SRFP1/4, FZD8/10, TCF7, and Wnt5a [48, 51–53, 56].

Details about the molecular markers analysis are reported for each marker in Table 2.

## 4. Discussion

This review investigated the efficacy and fertility outcomes of conservative treatment, also known as fertility-sparing treatment, in patients of reproductive age affected by EC stage IA G2. As fertility sparing treatment has become a viable option in case of early-stage EC, oncological and fertility outcomes have increasingly been investigated during the last decade. Even if some conservative methods have been proposed to preserve female fertility of patients with low grade and low stage of EC, the optimal management of these patients is still unknown. In particular, the experience with conservative treatment of stage IA G2 EC is very limited, partly due to the rarity of such a diagnosis in the reproductive age, partly due to the exclusion of these cases from fertility management. Currently, released guidelines for the conservative management of endometrial cancer focus on stage IA well-differentiated (G1) EEC, while stage IA moderately differentiated (G2) EEC is not yet included. Effectively, NCCN Guidelines perfectly outline the inclusion criteria in considering fertility-sparing options for management of EEC: G1 EEC on dilatation and curettage (D&C) confirmed by expert pathology review; disease limited to the endometrium (stage IA) on MRI (preferred) or transvaginal ultrasound [57]; absence of suspicious or metastatic disease on imaging; no contraindications to medical therapy or pregnancy; and patients who should undergo counseling that fertility-sparing option is not standard of care for the treatment of G1 EEC. Continuous progestin-based therapy, both oral progestins and LNG-IUDs, should be carefully evaluated, especially in the context of particular medical conditions. The guidelines also highlight in which patients is necessary to avoid this type of treatment such as breast cancer, stroke, myocardial infarction, pulmonary embolism, deep vein thrombosis, and smoking.

No consensus exists regarding which agent, dose, or duration of treatment is most effective. Anyway, although the histological grade changes, conservative treatments are similar. Generally, the most commonly employed agent is MPA at 400–600 mg daily or MA at 160–320 mg daily. In the last years, another employed agent is LNG-IUDs, associated or not with hysteroscopic resection, as described by Giampaolino et al. in EEC G1 stage IA and AEH. They have compared their results with oral hormonal therapy in a meta-analysis described by Wei et al and have shown a similar response and live birth rates but with a lower relapse rate (6,5% in Giampaolino et al. in comparison with 20% in Wei et al.) [29, 58]. Park et al. [45] conducted a multicenter retrospective cohort study showing that 37/48 patients (77.1%; 95% CI 63.3–86.9%) achieved CR with oral MPA or MA after the median treatment duration of 10 months (range 3–20 months). On multivariate analysis, the following variables, including progestin type, progestin dose, or duration of treatment, were not associated with a recurrence-free survival; on the contrary, a history of infertility (odds ratio 0.20, 95% CI 0.06–0.69; P5.011) and pregnancy (odds ratio 0.26, 95% CI 0.07–0.93; P5.038) were significantly associated to recurrence-free survival. Complete response rates were 76.5% (95% CI 52.2–91%), 73.9% (95% CI 53.2–87.7%), and 87.5% (95% CI 50.8–99.9%) for patients with stage IA G2–3 without myometrial invasion (*n* = 17), for patients with stage IA G1 with superficial myometrial invasion (*n* = 23), and for patients with stage IA G2–3 with superficial myometrial invasion (*n* = 8), respectively (*p* = .731). Any patient experienced disease progression or died from the disease. According to these data, the authors concluded that fertility-sparing treatment is safe in this group of patients.

On the other hand, Chae et al. [35] analyzed patients with G1-2 EEC undergoing fertility-sparing treatment and reported pregnancy outcomes. A total of 22/49 patients became pregnant, with a total of 30 pregnancies, of which 25 live births. The analyses for predicting pregnancy failure after fertility-sparing hormonal therapy demonstrated that a higher grade was also closely associated with pregnancy failure (OR 6.2, 95% CI 1.0 to 38.9; *p* < .05). Several studies have suggested that the plasminogen activator inhibitor type 1 level is higher in G2/3 than in G1 EEC, with a higher probability of thrombosis; so pregnancy failure might be related to a higher grade because of the higher PAI-1 level [59]. Effectively, Chae et al. reported that only 2/11 patients with EEC stage IA G2 became pregnant, compared to 19/37 patients with G1. The oncologic outcomes showed that patients who achieved pregnancy had recurrence later than the patients who did not conceive. The total recurrence rate was 36.7% (18/49). The pregnant and nonpregnant groups had a recurrence rate of 18.2% (4/22) and 51.9% (14/27), respectively (*p* < 0.05); the mean disease-free survival time was of 26 months (range 20–38) in the pregnant group opposed to 12 months (range 4–48) in the nonpregnant group (*p* < 0.05).

As regards the follow-up, in patients receiving progestin-based therapies, close monitoring with endometrial sampling (endometrial aspiration biopsy (EAB) or hysteroscopic biopsy or D&C) every 3 to 6 months is recommended. Patients should be advised in trying to conceive immediately (spontaneously or by ART) after complete response to the conservative treatment, if pregnancy is desired [60, 61]. Furthermore, TH with BSO and an accurate staging is indicated after childbearing, if patients have documented progression on the biopsies or in case of stable disease after 6 to 12 months of progestin-based therapy [62–64].

Related to the molecular aspect of EEC, several immunohistochemical markers and different pathways, potentially involved in a good response or resistance to progestin therapy, have been evaluated to predict response to treatment, even if their usefulness is still unclear. Numerous predictive molecular markers have been proposed: ER and, especially, PR are the most reported to date [46–48, 51, 53, 56]. The progestogens mediate their effects through PR, and the pathogenesis of EC presumes an imbalance between PR and ER. High expression of these receptors in pretreatment samples was predictive of a good response to conservative treatment, as shown by Chung et al. [53]; indeed patients with PR positivity showed a better response to treatment than patients with PR negativity (80.8% vs. 20.0%, *p* = 0.011). Meanwhile, no significant differences in response rates between patients with ER positivity and those with ER negativity (74.1% vs. 100.0%, *p* = 0.568) were found.

However, there is evidence that PR is not essential for response: actually, also PR-negative lesions can benefit from progestins. Moreover, a high level of the B isoform of PR (PRB) and ER are linked with a poorer prognosis during follow-up, as specified by Reyes et al. [48]. Effectively, PR and PRB levels should be decreased during progestin therapy, owing to short-term hormone receptor downregulation leading to desensitization to progestin [48, 65]. In patients with no progression, Reyes et al. identified a notable decrease in levels of ER, PR, and PRB in the posttreatment biopsies as compared to pretreatment, denoting that progestins attend ligand-mediated receptor downregulation. ER and PRB levels in the biopsies after IUD inserting were significantly higher in patients with progressive disease as compared to the other group, and the magnitude of the decrease in hormone receptor levels in posttreatment biopsies was significantly understated [48].

ER and PR status expression was not associated with treatment response, neither from Westin et al. [56] nor from Yang et al. [47]. Also Gunderson et al. discovered that the achievement of complete response was not associated with pre- and posttreatment ER expression (pretreatment ER percent: *p* = 1.0; ER intensity: *p* = 0.24-posttreatment ER percent: *p* = 0.53; ER intensity: *p* = 0.62). Even PR expression was unrelated to subsequent complete response: the posttreatment PR status was *p* = 0.47 and *p* = 1.0, respectively [46].

A recent meta-analysis conducted by Raffone et al. about immunohistochemical biomarkers for progestin response in women with endometrial hyperplasia or early EEC concluded that PR was a predictive biomarker only when intrauterine progestins were used [66].

Among poor response markers, recent evidence indicated the crucial role of the transcription factor NF-E2-related factor 2 (Nrf2). Fan et al., particularly, inquired if Ntrf2-survivin pathway contributes to progestin resistance [55]. A high level of protein expression was detected in endometrial tissue samples following treatment, highlighting a poor response to the progestin therapy, as previously investigated by Wang et al. [54]. On the contrary, all responded patients showed negative expression of these markers in the endometrial tissue samples, confirming the dysregulation of this pathway can play a possible role in progestin resistance in EC. Nrf2 and survivin expressions were suppressed after withdrawal of progestin [55].

Also AKR1C1 is involved in the Nrf2 pathway associated with progestogens resistance. Progestin withdrawal resulted in suppression of Nrf2/AKR1C1 expression, followed by a reduction of cellular proliferation [54]. Constitutive activation of the Wnt signaling pathway by genetic alterations of *β-catenin* genes has been observed in multiple malignancies. But yet, Van Gent et al. in their analysis have not found a relevant association between dysregulation of Wnt-*β-catenin* pathway and the lack of response to progesterone treatment.

Higher baseline expression of the proliferation marker Ki67 was associated with a poor response on pretreatment as well as deficiency of gene expression of Dickkopf-related protein 3 (DKK3) [56], while high level of Ki67 in posttreatment was associated with an increased risk of relapse [47]. Among DKK3, it may function as a tumor suppressor gene, in fact, its downregulation and methylation have been reported in many human cancers [67].

Sperm-associated antigen 9 (SPAG9), a recently characterized oncogene, was associated to the progression of several human cancer and previously correlated to the degree of differentiation and to lymphatic metastasis in ECC [68]. A lower expression of SPAG9 was detected in patients with better response to progestin, accompanied by decreasing level of SPAG9 in the endometrial tissue after conservative treatment. On the other side, the nonresponsive group, which includes resistance patients too, always manifested a SPAG9 upregulation [49]. Li et al. also specified how a better response was linked to AEH than ECC, and their results may therefore be more relevant in this field. Nowadays, it is possible to speculate its role as a marker for progestin resistance, but the following affirmative studies are needful to prove the role of SPAG9 in the endometrial cancer because of the small number of the cohort analyzed in this study.

Recently, Zhang et al. [69] evaluated the usefulness of dual-specific phosphatase 6 (Dusp6), a marker of the mitogen-activated protein kinase (MAPK) signaling pathway, to predict the response to progestogens in endometrial hyperplasia, showing that its high expression was predictive of good response, as well as in the pretreatment setting and during follow-up. In fact, the lack of expression of Dusp6 was an important sign of potential therapy failure, associated with poor response.

In a more recent study, Travaglino et al. [70] analyzed in detail the role of Dusp6 as a predictive marker of response of AEH and EEC in women undergoing to conservative treatment, highlighting that a weak expression of Dusp6, with moderate predictive accuracy, represents an important predictor of resistance of fertility-sparing treatment in AEH/EECs. Although both studies have obtained encouraging results about Dust6, as an important marker of response to progestin treatment, they have not been included in this review as Zhang et al. deals with only endometrial hyperplasia, while Travaglino et al. study is consecutive to the data sources, conducted from 2010 to September 2021.

MMR proteins, MLH-1, MSH-2, MSH-6, and PMS-2, are essential for repairing DNA errors, produced during DNA replication; in fact loss of function of one or more MMR proteins leads to impaired DNA repair capability, causing potential therapy failure, as reported by Zakhour and Chung et al. [50, 53]. Their analysis showed that deficiency of MMR was related with poor response in pretreatment setting. Moreover, Chung et al. did not report any statistically significant association with MMR in the follow-up [53].

## 5. Conclusion

We believe that the hormonal therapy, combined or not with hysteroscopic endometrial focal resection more evaluated in EEC G1 Stage 1A and AEH, could be considered an effective and safe approach in the management of EEC G2 Stage 1A in young women who desire to preserve fertility.

Furthermore, the different options that characterize the conservative management of EEC G2 stage IA should be compared to identify an optimal treatment and to improve fertility rates and pregnancy outcomes. However, long-term prognosis, including recurrence and survival rates, need to be further monitored. Another important aspect to take into account is the length of follow-up, too heterogeneous in the studies published so far.

The molecular categorization in a fertility-sparing setting is increasing, and data exposed are promising, PR and ER were the most studied predictive markers, in particular PR appeared as the most promising and showed a good response in case of high expression, in pretreatment. On the contrary, low levels of PR are linked to good response during follow-up. The deficiency of MMR was an important sign of potential therapy failure associated with poor response, as well as an overexpression of Nrf2-survivin pathway and of Ki67, that appeared as a significant predictor of progestin resistance in AEH/EEC. FOXO1 ssDNA, PTEN, beta catenin, p53, EIG121, IGF1/2, IGFBP1, SRFP1/4, FZD8/10, TCF7, and Wnt5a may also reasonably play a role, but today no significant association was found to the outcome of response to progestin therapy.

Considering markers of hormone response, actually, in literature, the greatest number of studies involves patients with EAH and G1 ECC. Future efforts should be focused on the utility of immunohistochemical markers in predicting hormone response and related outcomes in a fertility-sparing setting, also in patients with diagnosis of G2 ECC.

In conclusion, fertility-sparing treatment towards patients with EEC G2 stage IA needs further exploration by larger series and randomized clinical trials, to assess the effectiveness and safety of such combined treatments because, to date, the studies on which we are based on are retrospective and this represents a weakness for our results.

## Figures and Tables

**Table 1:** Characteristics of the studies and patients included in the review.

Studies	Countries	Study design	Number of cases	Age	Type of treatment
Koskas [39]	Paris, France	CR∗	Case 1 Case 2 Case 3	41 32 35	NES (20 mg/d) MA (160 mg/d) NG (5 mg/d)

Brown [40]	Houston, USA	CR∗	Case 1	18	LNG-IUDs (20 *μ*g/d)

Park [45]	Seoul, Korea	MRCS	14	(23-40)	MA (40-240 mg/d)/MPA (80-1000 mg/d)

Rossetti [41]	Bergamo, Italy	CR∗	Case 1 Case 2	(27–31)	MA (160 mg/d)

Kim [42]	Daegu, Korea	CR∗	Case 1	13	1°. MA (160 mg/d) 2°. MPA (10 mg/d)

Hwang [34]	Seoul, Korea	ROS	Case 1 Case 2 Case 3 Case 4 Case 5	28 25 29 31 39	MPA (500 mg/d) + LNG-IUDs (20 *μ*g/d)

Pal [43]	Houston, Texas	RCS	8	(18-85)	LNG-IUDs (20 *μ*g/d)

Chae [35]	Seoul, Korea	ROS	11	(28-45)	MPA (500 mg/d) + LNG-IUDs

Roberti Maggiore [36]	Milan, Italy	ROS	4	(31-37)	LNG-IUDs

Yu [37]	Beijing, China	ROS	8	(22-35)	3 MPA (500 mg/d) 3 MA (160 mg/d) 1 GnRHa (3,75 mg every 4 weeks) + AI + LNG-IUDs 1 MA(160 mg/d) → GnRHa (3,75 mg every 4 weeks) + LNG-IUDs

Falcone [44]	Naples, Italy	PS	23	(28-44)	12: HR + LNG-IUDs 4: HR + MA (160 mg/day) 4: LNG-IUDs 1: NES (10 mg/day) 1: LNG-IUDs + MA (160 mg/day) 1: MA (160 mg/day)

Shan [38]	Shanghai, China	ROS	Case 1 Case 2 Case 3 Case 4	28 38 35 36	MA (160 mg/day) + MET MA (160 mg/day) MA (160 mg/day), MA + MET, LNG-IUDs + GnRHa + MET, LNG-IUDs + GnRHa + LE + MET MA (160 mg/day) + MET

Total			84	13-85)	

AI: aromatase inhibitor; CR∗: case report; CR: complete response; GnRHa: gonadotropin-releasing hormone agonist; MA: megestrol acetate; MPA: medroxyprogesterone acetate; MRCS: multicenter retrospective cohort study; NES: norethisterone; NG: normegestrol; LE: letrozole; LNG-IUDs: levonorgestrel-releasing intrauterine devices; PS: prospective study; RCS: retrospective case series; ROS: retrospective observational study; SD: stable disease.

**Table 2:** Immunohistochemical markers assessed and their significant association found in the reviewed studies.

Studies	Immunohistochemical markers	Pretreatment				Treatment	Outcomes		Outcomes		Follow-up		
							Response		Relapse				
		Marker	Grade	Outcome	*p* value		Good	Poor	Yes	No	Marker	Outcome	*p* value
Kamoi [51]	Ki67 ssDNA ER PR	NA	G1IA	NA	NA	MPA	5	2	—	—	NA	NA	NA

Gunderson [46]	ER PR	NA	AEH G1	NA	NA	MA	30	16	7	23	NA	NA	NA

Yang [47]	ER PR Ki67	NA	AEH G1	NA	NA	MA MPA LNG-IUDs Norethisterone	77	11	25	46	↑ Ki67	R	0.033

Reyes [48]	FOXO1 ER PR PRB	NR	AEH G1	NA	NA	LNG-IUDs	7	3	—	—	↑ ER ↑ PRB ↓ ER ↓ PR ↓ PRB	PR PR GR GR GR	< 0.05 < 0.05 < 0.01 < 0.01 < 0.05

Van Gent [52]	PTEN *β*-Catenin	NA	G1IA	NA	NA	MA MPA MPA + LNG-IUDs	6	5	5	1	NR	NR	NR

Wang [54]	Nrf2 AKR1C1	—	NR	—	—	NR	11	10	—	—	↑ Nrf2 ↑AKR1C1	PR PR	<0.0001 <0.0001

Li [49]	SPAG9	NR	AEH G1IA	NR	NR	MPA	21	6	—	—	↓ SPAG9	GR	0.005

Fan [55]	Nrf2 Survivin	—	NR	—	—	MPA + metformin	18	17	—	—	↑ Nrf2 ↑ Survivin	PR PR	< 0.001 < 0.001

Zakhour [50]	MLH1 MSH2 MSH6 PMS2	MMR loss	AEH G1I A	PR	0.026	Oral progestin +/- LNG-IUDs Oral + injectable progestin	41	43	—	—	—	—	—

Chung [53]	P53 ER PR MLH1 MSH2 MSH6 PMS2	↓ MMR ↑ PR	G1 G2	PR GR	0.018 0.011	MPA MA MPA+ LNG-IUDs	43	14	19	24	NA	NA	0,069

Westin [56]	Ki67 DDK3 PR EIG121 IGF-1/2 IGFBP1 RALDH2 SRFP1/4 Survivin FZD8/10 TCF7 Wnt5a	↑ Ki67	AEH	PR	0.023	LNG-IUDs	37	10	4	33	NR	NR	NR
		↓ DDK3	G1	PR	0.030								

**Table 3:** Oncological and reproductive outcomes of the studies included in the review.

Studies	Number of cases	Surgery	Oncologic outcomes (CR, PR, SD) (months)	Oncologic outcomes (R) (months)	Pregnancies (number)	Live births	Follow-up (months)
Koskas [39]	Case 1 Case 2 Case 3	Refused — TH	CR (3) CR (6) CR (5)	EAG1 (6) — EAG1 (36)	0 1 0	NA Twins NA	AWD (12) NED (24) NED (60)

Brown [40]	Case 1	—	CR (3)	—	0	NA	NED (13)

Park [45]	14	NR	11 CR (3-12) 3 SD	3 (8-20)	3	NR	NED (7-136)

Rossetti [41]	Case 1 Case 2	TH after CS —	CR (6) CR (6)	— —	1 (IVF) 1 (IVF)	1 1	NED (14-52)

Kim [42]	Case 1	—	SD (3) CR (8)	—	0	NA	NED (8)

Hwang [34]	Case 1 Case 2 Case 3 Case 4 Case 5	— — — — TH/BSO	CR (9) CR (6) PR (12) CR (18) PR (9)	(23) — — — —	1 (IVF) 0 0 0 0	Abortion NA NA NA NA	NED (59) NED (19) NED (10) NED (55) NED (69)

Pal [43]	8	NR	3 CR (6) 3 PR (6) 2 SD (6)	NR	NR	NR	NR

Chae [35]	11	NR	NR	NR	2	NR	NR

Roberti Maggiore [36]	4	4	3 CR (4) 1PD (n/r)	3 (13-16)	NA	NA	NR (113-118)

Yu [37]	8	3 TH	7 CR (3-9) 1 SD/PD (9)	3 (17-36)	3	2 NFTD	NED (21-77)

Falcone [44]	23	6 TH (SD, PD) 1 TH (after CS) 6 TH (relapse) 1 TH (before the 5-years follow-up)	17 CR (6-13) 1 SD (6) 5 PD (3-12)	7 (4-142)	10 (2 ART)	3 NFTD 2 SFTM	22 NED 1 AWD (9-148)

Shan [38]	Case 1 Case 2 Case 3 Case 4	TH/BSO + PL (after 2.5 months) — — —	— CR (6) SD (3) SD (3) CR (3) CR (6)	— — (6) —	— Undergoing IVF Undergoing IVF Trying spontaneous pregnancy	— — — —	NED (52) NED (34) NED (7) NED (26)

Total	84	23	54 cr 6 sd 5 pr 7 pd 12 nr	20	22	8 nftd	59 NED 2 AWD 23 NR

ART: assisted reproduction technology; AWD: alive with disease; IVF: in vitro fertilization; NED: no evidence of disease; NFTD: normal full-term delivery; NA: not applicable; NR: not reported; CS: caeserean section; PL: pelvic lymphadenectomy; SFTM: spontaneous first-trimester miscarriage; TH/BSO: total hysterectomy+bilateral salpingo-oophorectomy; CR: complete response; PR: partial response; SD: stable disease; PD: progressive disease.

**Table 4:** Risk of bias assessment for case series using Joanna Brigg's critical appraisal tool for case series.

Studies	Item										Overall risk of bias
	1	2	3	4	5	6	7	8	9	10	
Koskas [39]	Y	NA	Y	N	N	Y	N	N	N	NA	High
Park [45]	Y	Y	Y	Y	Y	Y	Y	Y	Y	Y	Low
Rossetti [41]	Y	Y	Y	NA	NA	N	N	Y	U	NA	Unclear
Kim [42]	Y	NA	Y	NA	NA	NA	Y	Y	Y	NA	Low
Hwang [34]	Y	Y	Y	NA	Y	Y	Y	Y	Y	NA	Low
Chae [35]	Y	Y	Y	NA	Y	Y	Y	N	N	NA	Unclear
Pal [43]	Y	Y	Y	U	Y	Y	Y	U	U	Y	Low
Roberti Maggiore [36]	Y	Y	Y	Y	Y	Y	Y	Y	Y	NA	Low
Falcone [44]	Y	Y	Y	Y	Y	Y	Y	Y	Y	NA	Low
Yu [37]	Y	Y	Y	Y	Y	Y	Y	Y	Y	Y	Low
Brown [40]	Y	NA	Y	NA	Y	Y	Y	Y	Y	NA	Low
Shan [38]	Y	Y	Y	NA	Y	Y	Y	Y	Y	NA	Low

Evaluated items: (1) Were there clear criteria for inclusion in the case series? (2) Was the condition measured in a standard, reliable way for all participants included in the case series? (3) Were valid methods used for identification of the condition for all participants included in the case series? (4) Did the case series have consecutive inclusion of participants? (5) Did the case series have complete inclusion of participants? (6) Was there clear reporting of the demographics of the participants in the study? (7) Was there clear reporting of clinical information of the participants? (8) Were the outcomes or follow-up results of cases clearly reported? (9) Was there clear reporting of the presenting site(s)/clinic(s) demographic information? (10) Was statistical analysis appropriate? Available judgments for each supporting item were “yes” (Y), “no” (N), “unclear” (U), and “not applicable” (NA).

## Data Availability

Data are available on request.

## Abbreviations

EC: Endometrial cancer
EEC: Endometrioid endometrial cancer
AEH: Atypical endometrial hyperplasia
AI: Aromatase inhibitor
CR: Case report
CR: Complete response
GnRHa: Gonadotropin-releasing hormone agonist
MA: Megestrol acetate
MPA: Medroxyprogesterone acetate
MRCS: Multicenter retrospective cohort study
NES: Norethisterone
NG: Normegestrol
LE: Letrozole
LNG-IUDs: Levonorgestrel-releasing intrauterine devices
PD: Progressive disease
PR: Partial response
PS: Prospective study
RCS: Retrospective case series
ROS: Retrospective observational study
SD: Stable disease
ART: Assisted reproduction technology
AWD: Alive with disease
IVF: In vitro fertilization
NED: No evidence of disease
NFTD: Normal full-term delivery
PL: Pelvic lymphadenectomy
SFTM: Spontaneous first-trimester miscarriage
TH/BSO: Total hysterectomy+bilateral salpingo-oophorectomy.
